# Femoral pseudotumor secondary to injury in a patient with idiopathic thrombocytopenic purpura: Case report

**DOI:** 10.1097/MD.0000000000019788

**Published:** 2020-04-10

**Authors:** Tao Sun, Shu-Man Han, Wen-Juan Wu, Bu-Lang Gao

**Affiliations:** a Department of Orthopaedic Surgery; b Department of Radiology, The Third Hospital of Hebei Medical University, Shijiazhuang Hebei Province, China.

**Keywords:** idiopathic thrombocytopenic purpura, imaging, intraosseous hematoma, intraosseous pseudotumor, right femur

## Abstract

**Rationale:**

Idiopathic thrombocytopenic purpura (ITP) is the condition of having a low platelet count of unknown causes and is a poorly understood acquired hemorrhagic disease involving destruction of platelets in the reticuloendothelial system induced by antiplatelet antibodies. Patients with ITP can have traumatic intra-articular, intraosseous or soft tissue hemorrhage which may present as a rare intraosseous pseudotumor on medical imaging.

**Patient concerns:**

A 30-year old male patient had complaint of pain in the right leg for 1 year. Laboratory test revealed a much lower platelet count (3–12 × 10^9^/L).

**Diagnoses:**

Radiography and computed tomography showed expansive bone destruction in the distal segment of the right femur, and magnetic resonance imaging revealed heterogeneous signal intensity in the lesion. Lesion curettage and pathology showed an expansion cyst with a really thin cortical bone shell containing serum-like red liquid and some sediment-like deposit. Consequently, the diagnosis of a pseudotumor was confirmed.

**Interventions:**

Lesion curettage and bone graft surgery were performed, and 8 units of platelet were transfused to the patient. Giant cell reaction was found on the shell of the lesion, but no tumor cell was found on pathological examination.

**Outcomes:**

The platelet count was 308 × 10^9^/L 5 days after operation, and the clotting time was normal. At 6 month follow-up after lesion curettage, the patient remained normal with no deterioration in the lesion site.

**Conclusion:**

The diagnosis of a pseudotumor of ITP relies mainly on imaging findings of the lesion and, in particular, knowledge of the underlying bleeding disorders. Radiologist and pathologist should be aware of the characteristics of this rare complication of ITP and other bleeding disorders like hemophilia in order to avoid misinterpretation of the lesion as a tumor or infection disease.

## 1 Introduction

Idiopathic thrombocytopenic purpura (ITP) is the condition of having a low platelet count of unknown causes and is a poorly understood acquired hemorrhagic disease involving destruction of platelets in the reticuloendothelial system induced by anti-platelet antibodies.^1^,^2^ As most causes appear to be related to antibodies against platelets, ITP is also known as immune thrombocytopenic purpura or immune-mediated thrombocytopenic purpura. For many adult patients with ITP, only mild thrombocytopenia is present, and quite a few patients have no bleeding symptoms.^[3]^ These patients are only diagnosed with ITP when they have blood test revealing a low blood platelet count. However, a low platelet count in ITP can lead to a long time to clot or stop bleeding after injury.^[4]^ Occasionally, the patient with ITP can have traumatic intra-articular, intraosseous or soft tissue hemorrhage which may present as a rare intraosseous pseudotumor on medical imaging. In this paper, we described a rare case with ITP who had a pseudotumor in the distal segment of the right femur after sprain and discussed the mechanism for an intraosseous pseudotumor.

This study was approved by the ethics committee of the Third Hospital of Hebei Medical University, Hebei, China. Written informed consent was obtained from the patient for publication of the study and medical images.

## 2 Case presentation

Patient has provided informed consent for publication of the case. A 30-year old male patient presented to the orthopedics department with complaint of pain in the right leg for 1 year, with the pain being intermittent, aggravated on activities and relieved after rest. The patient had a history of ITP 15 years ago when the patient had repeated epistaxis, skin purpura, and other hemorrhage symptoms, which did not respond to glucocorticoids. However, after vincristine therapy, these symptoms were resolved for a long time. Two years ago, the patient sprained his right knee, but X-ray examination found nothing abnormal. One year later, he felt pain in his right leg, but nothing abnormal was found on physical examination. Laboratory test revealed a normal red blood cells and white blood cell count, but a much lower platelet count (3 × 10^9^/L to 12 × 10^9^/L, and the normal reference is 100 × 10^9^/L to 300 × 10^9^/L) with normal clotting time.

**Figure 1:**
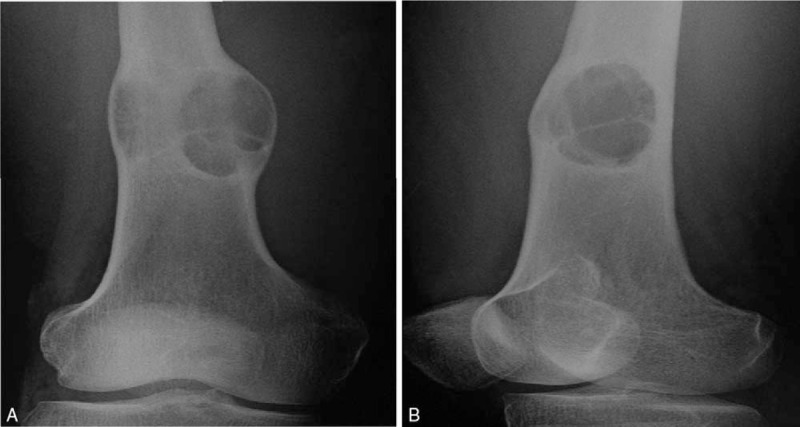
Radiography of the right femur. Anterioposterior (A) and oblique (B) radiograph of the femur revealed an expansive, well-defined radiolucent lesion in the distal segment of the right femur, with sclerotic rim and trabecula inside.

**Figure 2:**
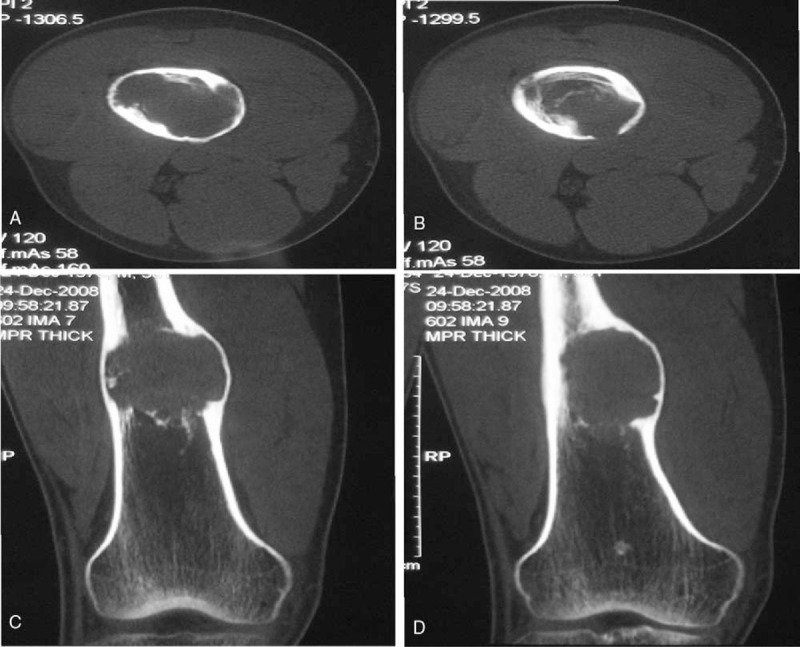
Computed tomography (CT) findings. Transaxial CT (A&B) and multiple plane reconstruction (C&D) demonstrated an expansive bone destruction region in the femur, with endosteal erosion, inner bone trabeculation, periosteal reaction, and posterial cortical disruption. Spotted calcification and “ground-glass-like” density are shown within the lesion. Average CT values in the lesion were 67-70Hu.

**Figure 3:**
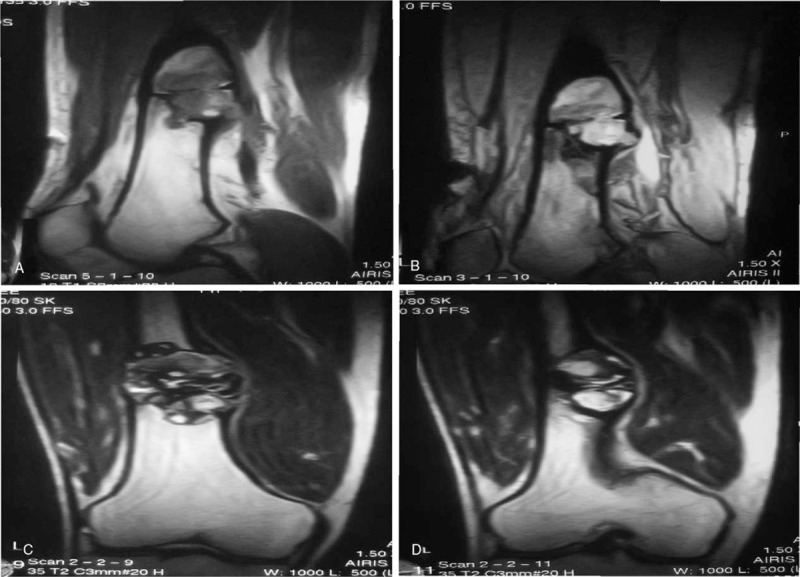
Magnetic resonance imaging of the lesion demonstrated heterogeneous signal intensity, with hypointensity and hyperintensity signal on T1 (A), T2 (B), and T2-weighted images (C&D).

Radiography of the right femur revealed an expansive, well-defined radiolucent lesion in the distal segment, with trabeculae inside and sclerotic margin (Fig. 1). Transaxial computed tomography and multiple plane reconstruction showed expansive bone destruction in the distal segment of the right femur, with endosteal erosion, inner bone trabeculation, periosteal reaction, and posterial cortical disruption (Fig. 2). Spotted calcification and “ground-glass-like” density were seen within the lesion. Magnetic resonance imaging revealed heterogeneous signal intensity in the lesion (Fig. 3), possibly reflecting different stages of blood degradation products, necrosis, calcification, and hemosiderin deposition. Based on the imaging findings, simple bone cyst, aneurysmal bone cyst, fibrous dysplasia, nonossifying fibroma, bone hemangioma, enchondroma, and even chondrosarcoma should be considered for the diagnosis. Chondroblastoma should also be considered if the lesion is adjacent to the joint.

**Figure 4:**
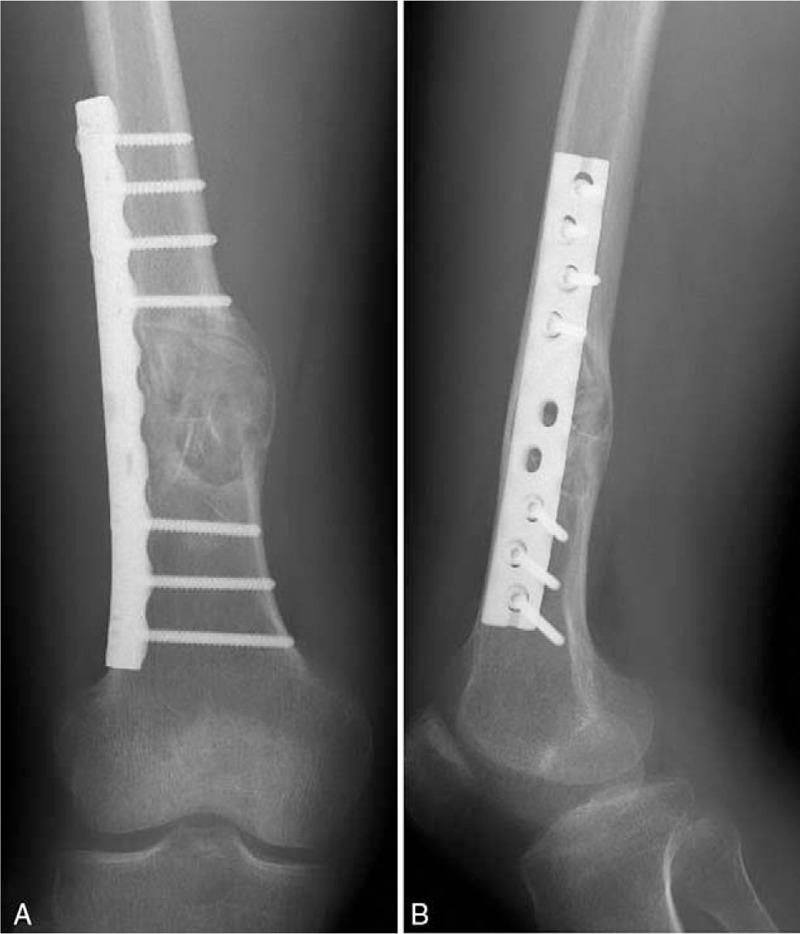
Post-operation radiography of the right femur. After lesion curettage, bone graft and steel plate fixation were performed.

**Figure 5:**
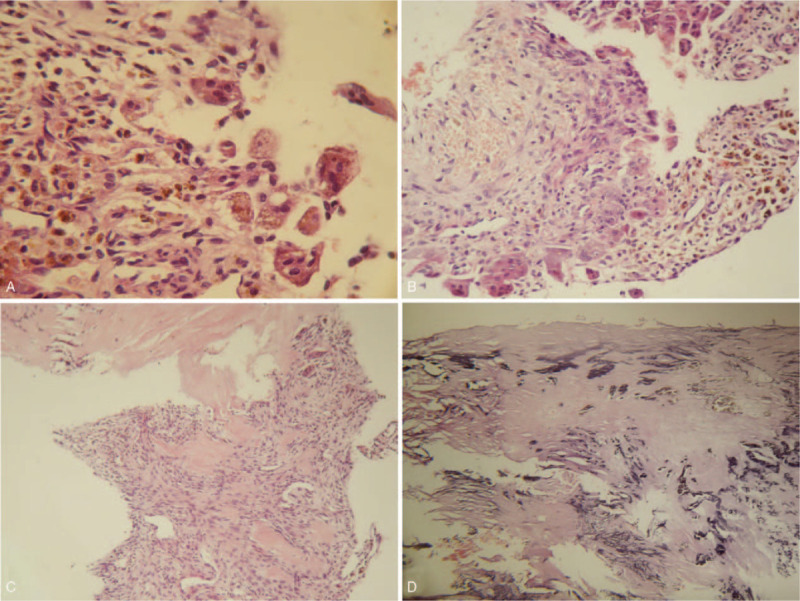
Hematoxylineosin staining of surgical curettage specimen (magnification was ×200, ×200, ×100 and ×200 for A, B, C, and D, respectively) was performed. A&B. The lesion had spindle fibrocyte proliferation, hemorrhage, hemosiderin deposition, and infiltration of inflammatory and multinucleated giant cells. C. Reactive new bone was formed in the background of spindle fibrocyte proliferation. D. Collagen degeneration and amorphous calcification were observed in the lesion.

The patient was treated with lesion curettage and bone graft surgery (Fig. 4). During the surgical procedure, the lesion was found to be an expansion cyst with a really thin cortical bone shell containing serum-like red liquid and some sediment-like deposit. Eight units of platelet were transfused to the patient during the operation to correct the low platelet count. The platelet count was 308 × 10^9^/L 5 days after operation, and the clotting time was normal. Giant cell reaction was found on the shell of the lesion, but no tumor cell was found on pathological examination (Fig. 5). No further ITP related therapy was performed. In the follow-up, the bone lesion began to heal.

Based on the clinical presentations, medical history, imaging and pathological findings, the final diagnosis was a pseudotumor. At 6 month follow-up, the patient remained normal with no deterioration in the lesion site.

## 3 Discussion

Since Starker reported the first cases of hemophilia pseudotumor of bone in 1918,^[5]^ cases of pseudotumor secondary to hemorrhagic diseases have been increasingly reported in the femur, tibia, fibula, and iliac bone.^6^,^7^,^8^,^9^,^10^,^11^ However, a pseudotumor of bone secondary to ITP was rare. The first case of pseudotumor secondary to ITP was reported in the fibula by Vilar et al in 1980,^[11]^ and 2 other cases were reported in the skull by Otsuka et al^[9]^ and in mandibula by Oda et al,^[8]^ respectively. In the report of the mandibular hemorrhagic bone cysts in a patient with ITP by Oda et al,^[8]^ the bone cyst in the left mandibula was secondary to a blow to the left mandible, causing possible bleeding within the mandibular bone. Our case was secondary to the right knee sprain and was initially misdiagnosed as an aneurysmal bone cyst because of the extremely low incidence of bone pseudotumor secondary to ITP which has similar X-ray findings.

Aneurysmal bone cyst is a benign bone lesion of expansion, like intraosseous cavities containing blood without endothelial membrane.^[12]^ It is most frequently found at proximal and distal metaphysis of the femur and the proximal metaphysis of tibia, but can occur in almost any bone segment in the body. Radiography showed a single translucent balloon-like lesion having a clear narrow sclerotic border and a “honeycomb” or “paliform” zone with irregular grid points inside.^[13]^ The intraosseous hematoma was excluded from diagnosis before operation because the case showed no symptoms of bleeding and coagulation disorders. Pathological diagnosis was aneurysmal bone cyst due to the nontypical pathological performance without taking into account of the ITP history.

Bone necrosis secondary to increased intraosseous pressure was the key pathophysiologic factor in the formation of a hemorrhagic bone pseudotumor. Silber et al^[10]^ suggested that bleeding under the periosteum and intraosseous would increase the pressure and finally lead to bone necrosis. Ghormley et al^[7]^ believed that a subperiosteal hematoma would stimulate new bone formation and further lead to bone destruction and absorption. However, intraosseous hematoma could cause pressure increase within the bone, and subsequent necrosis and resorption may bring about bone fracture or further bleeding, followed by new bone formation. There are some main factors to affect formation of a pseudotumor: pressure bone necrosis, new bone formation, continuous bleeding, and hematoma organization.^[6]^ A slight trauma of the femur may lead to symptoms on the knee rather than the hip at a very early stage, and if subperiosteal hemorrhage occurred, a pseudotumor of bone would form several weeks or years later. In soft tissues, bleeding may lead to formation of a hematoma, increase the tissue pressure, and finally cause compartment syndrome. But if the bleeding is subperiosteal or intraosseous, pressure bone necrosis, and resorption may develop followed by calcification of the hematoma. The range of bone destruction is related to the pressure caused by the hematoma, and high pressure at the early stage may involve a large area. An obsolete intraosseous pseudotumor usually presents with an expansion bone destruction area displaying variant degrees of calcification in the center and small bone column formation on the border, and may further induce cortical bone formation and repair, especially in well differentiated pseudotumors. For our patient, we presumed that the right knee sprain induced intraosseous bleeding in his femur and eventual formation of the intraosseous pseudotumor. Significantly lower blood platelet count led to prolonged bleeding but normal coagulation time. Although the patients disease was stable for many years, his platelet count was very low, which could cause bleeding and local hematoma formation in the body when a subtle trauma occurred as seen in this patient.

Bone reaction caused by intraosseous hematoma is similar to giant cell reparative granuloma histologically. Indeed, a giant cell reparative granuloma of bone is a response to intraosseous hemorrhage.^[14]^ Furthermore some examples of the solid type of aneurysmal bone cyst are considered to be giant cell reparative granulomas.^[15]^ Moreover, an aneurysmal bone cyst is non-neoplastic in nature and may resemble reactive processes like a giant cell reparative granuloma and hyperparathyroidism radiographically and histologically.

In our case, a correct diagnosis could not be reached based only on imaging presentations because similar imaging presentations could be found in cases with simple bone cyst, aneurysmal bone cyst, fibrous dysplasia, nonossifying fibroma, bone hemangioma, enchondroma, chondrosarcoma, and even chondroblastoma. The correct diagnosis was only made during open surgery with histopathological examination of the excised sample of the lesion. In curettage and bone graft surgery of the lesion, it was found to be an expansion cyst with a really thin cortical bone shell containing serum-like red liquid and some sediment-like deposit. Giant cell reaction was found on the shell of the lesion, but no tumor cell was found on pathological examination. A pseudotumor was diagnosed according to clinical presentations, medical history, imaging, and pathological findings.

In conclusion, the diagnosis of a pseudotumor of ITP relies mainly on the imaging findings of the lesion and, in particular, knowledge of the underlying bleeding disorders. Radiologist and pathologist should be aware of the characteristics of this rare complication of ITP and other bleeding disorders like hemophilia in order to avoid misinterpretation of the lesion as a tumor or infection disease.

## Author contributions

**Conceptualization:** Wen-Juan Wu, Bu-Lang Gao.

**Data curation:** Tao Sun, Shu-Man Han.

**Formal analysis:** Wen-Juan Wu.

**Investigation:** Tao Sun, Shu-Man Han.

**Methodology:** Tao Sun, Shu-Man Han.

**Project administration:** Tao Sun, Shu-Man Han.

**Resources:** Wen-Juan Wu.

**Supervision:** Shu-Man Han, Wen-Juan Wu.

**Validation:** Bu-Lang Gao.

**Writing – original draft:** Tao Sun.

**Writing – review & editing:** Bu-Lang Gao.
